# Association of nutritional status-related indices and chemotherapy-induced adverse events in gastric cancer patients

**DOI:** 10.1186/s12885-016-2934-5

**Published:** 2016-11-18

**Authors:** Seung Hee Seo, Sung-Eun Kim, Yoon-Koo Kang, Baek-Yeol Ryoo, Min-Hee Ryu, Jae Ho Jeong, Shin Sook Kang, Mihi Yang, Jung Eun Lee, Mi-Kyung Sung

**Affiliations:** 1 Department of Food and Nutrition, Sookmyung Women’s University, 100, Cheongpa-ro 47-gil, Yongsan-gu, Seoul, 04310 South Korea; 2 Department of Oncology, Asan Medical Center, University of Ulsan College of Medicine, Seoul, 05505 South Korea; 3 Department of Dietetics and Nutrition Services Team, Asan Medical Center, Seoul, 05505 South Korea; 4 Research Center for Cell Fate Control, College of Pharmacy, Sookmyung Women’s University, Seoul, 04310 Republic of Korea

**Keywords:** Gastric cancer, Malnutrition, Chemotherapy, Adverse events, Hypoalbuminemia

## Abstract

**Background:**

Malnutrition in gastrectomized patients receiving chemotherapy is associated with the susceptibility to chemotherapy-related adverse events. This study evaluated pre-operative nutritional status-related indices associated with adverse events in post-operation gastric cancer patients receiving chemotherapy.

**Methods:**

Medical records of 234 gastrectomized patients under adjuvant tegafur/gimeracil/oteracil chemotherapy with extended lymph node dissection were analyzed. Nutritional status assessment included Patient-Generated Subjective Global Assessment (PG-SGA), body weight, body mass index, serum albumin concentration, and Nutrition Risk Index (NRI). Chemotherapy-originated adverse events were determined using Common Terminology Criteria for Adverse Events.

**Results:**

PG-SGA indicated 59% of the patients were malnourished, and 27.8% of the patients revealed serious malnutrition with PG-SGA score of ≥9. Fifteen % of patients lost ≥10% of the initial body weight, 14.5% of the patients had hypoalbuminemia (<3.5 g/dL), and 66.2% had NRI score less than 97.5 indicating moderate to severe malnutrition. Hematological adverse events were present in 94% (≥grade 1) and 16.2% (≥grade 3). Non-hematological adverse events occurred in 95.7% (≥grade1) and 16.7% (≥grade 3) of the patients. PG-SGA and NRI score was not associated with treatment-induced adverse events. Multivariate analyses indicated that female, low body mass index, and hypoalbuminemia were independent risk factors for grade 3/4 hematological adverse events. Age was an independent risk factor for grade 3/4 non-hematological adverse events. Neutropenia was the most frequently occurring adverse event, and associated risk factors were female, total gastrectomy, and hypoalbuminemia.

**Conclusions:**

Hypoalbuminemia, not PG-SGA or NRI may predict chemotherapy-induced adverse events in gastrectomized cancer patients.

**Electronic supplementary material:**

The online version of this article (doi:10.1186/s12885-016-2934-5) contains supplementary material, which is available to authorized users.

## Background

Malnutrition in cancer patients originates from tumor-induced metabolic abnormalities and treatment-related side effects [1]. Previous studies suggested that 40-80% of cancer patients experience malnutrition which is also a major cause of cancer deaths [2, 3]. Gastrectomy followed by chemotherapy is the mainstay to treat gastric cancer, and majority of gastric cancer patients under treatment experiences malnutrition. Partial or full gastrectomy reduces food intake per serving and induces anastomosis and vagal block causing abdominal sense of distension, discomfort, and frequent bowel movement. Chemotherapy following gastrectomy also induces anorexia, sore throat, dry mouth, taste change, nausea, diarrhea, constipation, and fatigue which eventually lead to weight loss and malnutrition [4]. High risk of malnutrition among gastrectomy patients was shown to delay the rate of recovery and increase cancer deaths [5]. Malnutrition in cancer patients reduces responses to the treatment [6] and increases treatment-associated side effects [7] often lowering the intensity of treatment protocol and sometimes treatment withdrawal. Therefore, it is important to determine nutritional status of gastric cancer patients before starting chemotherapy to maximize the efficiency of treatment as well as the patients’ quality of life.

Nutrition screening is an important step to identify cancer patients who are at risk for malnutrition and to provide information required for treatment protocol preparation. The Malnutrition Universal Screening Tool (MUST) scores are often used for surgical patients and have been validated for cancer patients [8]. However, the applicability and accuracy of MUST in assessing nutritional status of gastric cancer patients has not been well evidenced. Patient-Generated Subjective Global Assessment (PG-SGA) is the most frequently used nutritional assessment tool for cancer patients [9]. It is a comprehensive screening method comprised of weight loss, performance status scores, and clinical evaluation scores recommended for assessing malnutrition in surgical cancer patients. A number of studies have also suggested that serum albumin concentration is a mortality prognosis factor in cancer patients [10]. Nutrition Risk Index (NRI) is another tool to assess nutritional status of operated patients receiving parenteral nutrition using serum albumin and weight change [11].

The standardized treatment protocol for stage 2 or 3 stomach cancer is gastrectomy followed by adjuvant tegafur/gimeracil/oteracil (S-1) chemotherapy [12]. Although S-1 exhibits relatively mild side effects, it has been reported that the completion rate at 1 year after gastrectomy is 65.8 and 42.4% of the patients need dose modification mostly due to treatment-associated side effects [12]. Age over 65 and total gastrectomy have been suggested as risk factors associated with treatment-associated side effects in these patients [13]. In the present study, we identified baseline nutritional status-related indices most closely related to the chemotherapy-induced adverse events in stage 2 and 3 gastrectomy cancer patients.

## Methods

### Study patients

This is a retrospective study using medical records of the patients diagnosed as having stomach cancer from Asan Medical Center in Seoul, Korea between October, 2007 and December, 2009. A total of 305 patients were screened and those with stage 2 or 3A stomach cancer according to the Guideline of the American Joint Committee on Cancer, and records of 234 gastrectomy patients (≥20 years old) who underwent S-1 chemotherapy and who had 0 to 2 physical performance level based on the guideline of the Eastern Cooperative Oncology Group were included. S-1 chemotherapy was performed within 3 to 6 weeks after gastrectomy in most of the patients. Patients who had experience of cancer therapy, need different therapy protocols, had other types of diseases including liver diseases and kidney diseases, transferred to other hospitals, and had mental illness were excluded. The study was approved by the Institutional Review Board of Asan Medical Center (2012–0221).

### Treatment protocol and measurement of adverse events

Treatment schedule of S-1 is composed of a 40 mg dosage/m^2^ body surface area twice a day for 4 weeks followed by a 2 weeks of off period. Patients received a maximum of 8 cycles of S-1 treatment for a year. Once there are hematological adverse events (≥grade 3) or non-hematological adverse events (≥grade 2), the dosage was reduced by the discretion of the doctor. Treatment associated adverse events were determined and recorded by the guidelines of Common Terminology Criteria for Adverse Events (CTCAE, version 3) which are based on hematological tests, radiological examinations, and physical examinations. The score of each side effect was the highest score among measurements during the treatment period. Changes in drug dosage and suspension of ongoing therapy due to side effects were also recorded with respective reasons. Hematological examinations were performed before starting a cycle of 6 weeks treatment. Recurrence was determined the 2^nd^ or 3^rd^ abdominal CT and endoscopy at the end of the final treatment cycle.

### Clinical assessments

Age, sex, tumor stage, height and weight, extent of surgery, medical history, treatment compliances, creatinine clearance, drug dosage, and existence of recurrence were examined prior to the first S-1 chemotherapy. It was reported that patients with kidney malfunction that fail to remove S-1 metabolites resulted in severe side effects [14].

### Nutritional assessments

Scores of PG-SGA and NRI determined before starting the first cycle of chemotherapy were used. PG-SGA scores were composed of two sections of numerical scores. The first section was a medical history section completed by the patient, and the second section was a physical examination section completed by medical staff. The sum of scores were grouped as A (<9, mild or moderate malnutrition) and B (≥9, severe malnutrition). NRI was calculated as follows: NRI = (1.519 × serum albumin, g/dL) + {41.7 × present weight (kg)/ideal body weight (kg)}. Nutritional risk was defined as three grades: 1) major risk (NRI < 83.5); 2) moderate risk (NRI 83.5 ~ 97.5); 3) mild risk (NRI 97.5 ~ 100). Body weight change between admission and the start of first treatment cycle was calculated and Body Mass Index (BMI) was determined.

### Statistical analyses

Statistical analyses were performed using SPSS version 18.0 (Chicago, IL, USA). Demographical and clinical data were allocated into either continuous data or categorical data. Continuous data were expressed as means ± standard deviations while categorical data were expressed as absolute or relative frequencies. Associations between adverse event and clinical or nutritional variables were analyzed by binary logistic regression analyses. Covariates used in each multivariate analysis were those which showed significance in univariate analysis.

## Results

### Patient characteristics and dietary regimen

Clinical characteristics of the patients are presented in Table 1. Study subjects were composed of 147 males and 87 females. The median age was 55 years old, and there were 38 patients who were over 65 years old. The number of stage 2 and 3A patients were 123 and 120, respectively. The number of patients who underwent total gastrectomy was 93, and the rest of the patients underwent partial gastrectomy. Patients were hospitalized for a week after gastrectomy as a standard procedure. During their stay in hospital, post-operative therapeutic diet accompanied by oral nutritional supplement (ONS) were provided. At the time of discharge, the patients were also advised to use ONS at home.

**Table 1:** Characteristics of the patients

Characteristic	No (%) (*n* = 234)
Sex	
Male	147 (62.8)
Female	87 (37.2)
Age (yr)	
Median	55
Range	25–78
Tumor stage	
T1^a)^	4 (1.70)
T2a	56 (23.9)
T2b	118 (50.4)
T3	56 (23.9)
Nodal stage	
N0^b)^	17 (7.3)
N1	137 (58.5)
N2	80 (34.2)
Cancer stage, TNM^c)^ classification	
II	123 (50.6)
III	120 (49.4)
Type of gastrectomy	
Total	93 (39.7)
Distal	141 (60.3)

^a)^T, the extent of the primary tumor; ^b)^N, the absence or presence and extent of regional lymph node metastasis; ^c)^The TNM system is an expression of the anatomic extent of disease and is based on the assessment of T, N, M. M-The absence or presence of distant metastasis

### Evaluation of nutritional status-related indices

Results of nutritional status-related assessments are shown in Table 2. The average weight loss between the first admission for operation and starting point of the first cycle of the chemotherapy was 6.8%. There were 36 patients (15.4%) who lost ≥10% of their body weight. At the starting point of the chemotherapy, 27 patients (11.5%) were underweight (BMI < 18.5 kg/m^2^) and 14.5% of the patients were hypoalbuminemia (<3.5 g/dL). Patients with PG-SGA category B were 59.0 and 27.8% of the patients exhibited PG-SGA score ≥9 which indicates the need for aggressive nutrition intervention. Assessment results from NRI showed that 63.2% of the patients were moderate malnutrition while 3.0% of the patients belong to the severe malnutrition group.

**Table 2:** Nutritional status-related indices of the patients

Index	No (%) (*n* = 234)
Weight loss (%)	
mean	6.8
<10% weight loss	198 (84.6)
≥10%	36 (15.4)
Body mass index (kg/m^2^)	
mean	21.86
<18.5	27 (11.5)
≥18.5	207 (88.5)
Albumin (g/dL)	
mean	3.71
<3.5	34 (14.5)
≥3.5	200 (85.5)
PG-SGA category	
A	96 (41.0)
B	138 (59.0)
PG-SGA score	
mean	7.42
<9	169 (72.2)
≥9	65 (27.8)
NRI	
mean	95.2
>97.5	79 (33.8)
83.5–97.5	148 (63.2)
<83.5	7 (3.0)

*PG-SGA* Patient-Generated Subjective Global Assessment, *NRI* Nutritional Risk Index

### Treatment-associated adverse events

Table 3 indicates hematological and non-hematological adverse events reported in study subjects. Ninety-four percent of the subjects experienced hematological adverse events with > grade 1, and 16.2% had adverse events greater than grade 3. The most common adverse event among those over grade 3 was neutropenia; 32 (13.6%) and 7 (3%) patients exhibited neutropenia and anemia, respectively. Non-hematological adverse events with > grade 1 were present in 95.7% of the patients. The patients who experienced non-hematological adverse events greater than grade 3 were 16.7%. The most common adverse event was diarrhea followed by abdominal pain. There was no cancer-related death. Seventy percent of the patients finished their therapy as it was planned. However, 27.4% of the patients were subjected to drug dose reduction and 13.6% of the patients needed to cease the therapy (data not shown). We found that age was positively associated with completion of chemotherapy (OR 1.04; 95% CI 1.01–1.07, *p* = 0.003; Additional file 1: Table S1). In addition, there was a positive relationship of total gastrectomy (OR 2.20; 95% CI 1.15–4.23, *p* = 0.018) and hypoalbuminemia (OR 2.25; 95% CI 1.01–5.04, *p* = 0.048) with delayed chemotherapy (Additional file 1: Table S1). We did not observe any significant relationship of PG-SGA or NRI with either completion or delay of chemotherapy. The common adverse events such as diarrhea and abdominal pain were not significantly associated with PG-SGA or NRI (Additional file 2: Table S2).

**Table 3:** Hematological and non-hematological adverse events (*n* = 234)

Events		Grade of chemotherapy toxicity (No (%))					
		Grade 0	Grade 1	Grade 2	Grade 3	Grade 4	Grade 3 + 4 (%)
Hematological adverse events	Anemia	29(12.4)	153(65.4)	45(19.2)	7(3.0)	–	3
	Neutropenia	94(40.2)	41(17.5)	67(28.6)	31(13.2)	1(0.4)	13.6
	Thrombocytopenia	150(64.1)	73(31.2)	10(4.3)	1(0.4)	–	0.4
	Febrile neutropenia	233(99.6)	–	–	1(0.4)	–	0.4
	Leucopenia	125(53.4)	86(36.8)	22(9.4)	1(0.4)	–	0.4
	Total	14(6.0)	95(40.6)	87(37.2)	37(15.8)	1(0.4)	16.2
Non-hematological adverse events	Anorexia	142(60.7)	61(26.1)	28(12.0)	3(1.3)	–	1.3
	Nausea	165(70.5)	51(21.8)	17(7.3)	1(0.4)	–	0.4
	Vomiting	206(88.0)	16(6.8)	11(4.7)	1(0.4)	–	0.4
	Diarrhea	119(50.9)	58(24.8)	43(18.4)	14(6.0)	–	6
	Constipation	213(91.0)	19(8.1)	2(0.9)	–	–	0
	Abdominal pain	183(78.2)	31(13.2)	10(4.3)	10(4.3)	–	4.3
	Stomatitis	191(81.6)	26(11.1)	15(6.4)	2(0.9)	–	0.9
	Hand-foot syndrome	200(85.5)	27(11.5)	6(2.6)	1(0.4)	–	0.4
	Fatigue	194(82.9)	35(15.0)	4(1.7)	1(0.4)	–	0.4
	Hyperpigmentation	183(78.2)	47(20.1)	3(1.3)	1(0.4)	–	0.4
	Rash	215(91.9)	14(6.0)	3 (1.3)	2(0.9)	–	0.8
	Elevated AST/ALT level	149(63.7)	77(32.9)	5(2.1)	3(1.3)	–	1.3
	Elevated bilirubin level	89(38.0)	99(42.3)	43(18.4)	3(1.3)	–	1.3
	Elevated creatinine level	230(98.3)	4(1.7)	–	–	–	0
	Total	10(4.3)	99(23.1)	86(36.8)	39(16.7)	–	16.7

*AST/ALT* aspartate transaminase/alanine transaminase

Binary logistic regression analyses indicated that female sex, hypoalbuminemia, and BMI were associated with adverse event greater than grade 3 (Table 4). Multivariate analyses showed the hypoalbuminemia (OR 2.45; 95% CI 1.02–5.89, *p* = 0.045) and female sex (OR 2.39; 95% CI 1.15–4.96, *p* = 0.020) were significant risk factors while BMI was a protective factor (OR 0.86; 95% CI 0.74–0.99, *p* = 0.035) (Table 4). Clinical and nutritional assessment indices related to non-hematological adverse events are shown in Table 5. Age was associated with adverse events greater than grade 3. Multivariate analyses indicated that age was a significant risk factor (OR 1.06; 95% CI 1.03–1.10, *p* < 0.001) (Table 5). There was no significant relationship of PG-SGA and NRI with hematological or non-hematological adverse events (Tables 4 and 5).

**Table 4:** Multivariable logistic regression analysis of risk factors for hematological adverse events

Variables		Grade 0–2 (*N* = 196)	Grade 3–4 (*N* = 38)	Univariate		Multivariate ^a)^	
				OR (95% CI)	*p*	OR (95% CI)	*p*
Age (y)				0.99 (0.96–1.02)	0.472		
Sex	Female	66 (75.9%)	21 (24.1%)	2.43 (1.20–4.92)	0.013	2.39 (1.15–4.96)	0.020
	Male	130 (88.4%)	17 (11.6%)	1.00		1.00	
Operation type	Total	72 (77.4%)	21 (22.6%)	2.13 (1.05–4.29)	0.035	1.79 (0.86–3.74)	0.119
	Distal	124 (87.9%)	17 (12.1%)	1.00		1.00	
Creatinine clearance (mL/min) ^b)^	≥60	165 (83.8%)	32 (16.2%)	1.00	0.997		
	<60	31 (83.8%)	6 (16.2%)	0.99 (0.39–2.59)			
Albumin (g/dL)	<3.5	24 (70.6%)	10 (29.4%)	2.56 (1.11–5.92)	0.028	2.45 (1.02–5.89)	0.045
	≥3.5	172 (86.0%)	28 (14.0%)	1.00		1.00	
BMI (kg/m^2^)				0.83 (0.72–0.95)	0.007	0.86 (0.74–0.99)	0.035
PG-SGA category	A	78 (81.3%)	18 (18.8%)	1.00	0.078		
	B	118 (85.5%)	20 (14.5%)	0.44 (0.17–1.09)			
PG-SGA score				0.88 (0.76–1.01)	0.083		
Weight loss (%)				0.97 (0.88–1.07)	0.558		
NRI				0.96 (0.89–1.02)	0.163		

^a)^Covariates used in multivariate analyses included sex, operation type, albumin, and BMI; ^b)^Creatinine clearance was calculated using the Cockroft–Gault formula; *BMI* Body Mass Index, *PG-SGA* Patient-Generated Subjective Global Assessment, *NRI* Nutritional Risk Index

**Table 5:** Multivariable logistic regression analysis of risk factors for non-hematological adverse events

Variables		Grade 0–2 (*N* = 195)	Grade 3–4 (*N* = 39)	Univariate		Multivariate ^a)^	
				OR (95% CI)	*p*	OR (95% CI)	*p*
Age (y)				0.83 (0.72–0.95)	0.007	1.06 (1.03–1.10)	0.000
Sex	F	72 (82.8%)	15 (17.2%)	1.07 (0.53–2.17)	0.856		
	M	123 (83.7%)	24 (24.55)	1.00			
Operation type	Total	78 (83.9%)	15 (16.1%)	0.94 (0.46–1.90)	0.858		
	Distal	117 (83.05)	24 (17.0%)	1.00			
Creatinine clearance (mL/min) ^b)^	≥60	170 (86.3%)	27 (13.7%)	1.00	0.007	1.00	0.370
	<60	25 (67.6%)	12 (32.4%)	3.02 (1.36–6.72)		1.52 (0.61–3.79)	
Albumin (g/dL)	<3.5	25 (73.5%)	9 (26.5%)	2.04 (0.87–4.04)	0.102		
	≥3.5	170 (85.0%)	30 (15.0%)	1.00			
BMI (kg/m^2^)				0.97 (0.85–1.10)	0.581		
PG-SGA category	A	84 (87.5%)	12 (12.5%)	1.00	0.157		
	B	111 (80.4%)	27 (19.6%)	1.70 (0.82–3.56)			
PG-SGA score				1.03 (0.91–1.17)	0.658		
Weight loss (%)				0.96 (0.86–1.07)	0.442		
NRI				0.95 (0.89–1.01)	0.083		

^a)^Covariates used in multivariate analyses included age and creatinine clearance; ^b)^Creatinine clearance was calculated using the Cockroft–Gault formula; *BMI* Body Mass Index, *PG-SGA* Patient-Generated Subjective Global Assessment, *NRI* Nutritional Risk Index

Binary logistic regression analyses between neutropenia and clinical or nutritional variables suggested that female sex, total gastrectomy, and hypoalbuminemia were risk factors for neutropenia (Table 6). Multivariate analyses indicated that female sex (OR 2.24; 95% CI 1.03–4.87, *p* = 0.043), total gastrectomy (OR 2.71; 95% CI 1.23–5.97, *p* = 0.013) and hypoalbuminemia (OR 2.61; 95% CI 1.01–6.54, *p* = 0.045) were independent risk factors (Table 6). No significant relationship of PG-SGA or NRI with neutropenia was observed (Table 6).

**Table 6:** Multivariable logistic regression analysis of risk factors for neutropenia

Variables		Grade 0–2 (*N* = 202)	Grade 3–4 (*N* = 32)	Univariate		Multivariate ^a)^	
				OR (95% CI)	*p*	OR (95% CI)	*p*
Age (y)				0.99 (0.42–2.33)	0.554		
Sex	F	70 (80.5%)	17 (19.5%)	2.14 (1.01–4.54)	0.048	2.24 (1.03–4.87)	0.043
	M	132 (89.8%)	15 (10.2%)	1.00		1.00	
Operation type	Total	73 (78.5%)	20 (21.5%)	2.95 (1.36–6.37)	0.006	2.71 (1.23–5.97)	0.013
	Distal	129 (91.5%)	12 (8.5%)	1.00		1.00	
Creatinine clearance (mL/min) ^b)^	≥60	170 (86.3%)	27 (13.7%)	1.00	0.975		
	<60	32 (86.5%)	5 (13.5%)	0.98 (0.35–2.75)			
Albumin (g/dL)	<3.5	25 (73.5%)	9 (26.5%)	2.77 (1.15–6.66)	0.023	2.61 (1.01–6.54)	0.040
	≥3.5	177 (88.5%)	23 (11.5%)	1.00		1.00	
BMI (kg/m^2^)				0.87 (0.75–1.00)	0.054		
PG-SGA category	A	80 (83.3%)	16 (16.7%)	1.00	0.106		
	B	122 (88.4%)	16 (11.6%)	0.44 (0.16–1.19)			
PG-SGA score				0.87 (0.75–1.02)	0.080		
Weight loss (%)				1.11 (0.92–1.12)	0.728		
NRI				0.95 (0.89–1.01)	0.110		

^a)^Covariates used in multivariate analyses included sex, operation type, and albumin; ^b)^Creatinine clearance was calculated using the Cockroft–Gault formula; *BMI* Body Mass Index, *PG-SGA* Patient-Generated Subjective Global Assessment, *NRI* Nutritional Risk Index

## Discussion

This study was conducted to identify sensitive nutritional status-related indices associated with the appearance of chemotherapy-induced adverse events in patients who underwent gastrectomy. S-1 complementary chemotherapy is a part of standard treatment protocol for stage 2 and 3A stomach cancer patients. Gastrectomy patients receiving S-1 therapy can start oral feeding within 6 weeks while the transition period from nutritional support to oral feeding is normally 6 months in these patients experiencing a great deal of weight loss. Few studies have provided evidences showing association between pretreatment nutritional status indices and treatment-associated adverse events which possibly delay the completion of treatment and the recovery.

The rates of adverse events observed in our subjects under S-1 therapy were similar to those reported in the previous study with S-1 gastrectomy patients [12]. Most frequent hematological and non-hematological adverse events were neutropenia and diarrhea. Seventy % of 234 patients completed the therapy according to the therapy protocol, and 27.4% of the patients were subjected to dosage reduction and 12.6% discontinued the therapy indicating at least one third of the gastrectomy patients did not tolerate S-1 therapy. Study results clearly suggested that patients with hypoalbuminemia and low BMI are highly susceptible to hematological adverse events and neutropenia was specifically frequent among patients with hypoalbuminemia. S-1 therapy usually starts 3 to 6 weeks after gastrectomy operation when operation-related fluctuation of other biochemical indices become stable and there is less chance of albumin being affected by operation itself. Therefore, the circulating albumin concentration can partly predict the compliance with adjuvant chemotherapy.

A meta-analysis study has suggested that serum albumin is a reliable index to predict cancer survival [10]. Moreover, hypoalbuminemia was reported as an independent prognostic marker of cancer fatality [15], and prognostic nutritional index calculated based on serum albumin and lymphocytes was reported as a prognostic marker in stomach cancer patients [16].

The increased synthesis of IL-1β, IL-6 and TNF-α is known to suppress liver synthesis of albumin, and the extent of reduction is related to disease severity [17, 18]. The increased cytokine concentration is a hallmark of cancer cachexia accelerating tumor-associated tissue wasting and anorexia [19] and thus, decreases in albumin concentration may well reflect cancer cachexia. Given that other physiological factors including liver cirrhosis, nephritic syndrome, catabolic status, blood dilution, and decreased removal rate of lymphocytes can also induce changes in serum albumin concentration [20], hypoalbuminemia as an accurate index of nutritional status in cancer patients has been criticized [21]. Other studies have proposed that there is an increase in vascular permeability of albumin accelerating albumin flux towards extravascular compartment [22] and the disruption of blood albumin homeostasis due to an increase in albumin degradation [23].

Despite the importance of nutritional status of patients receiving cancer treatment, a limited number of studies evaluated the association between nutritional status and other related indices. A previous study reported that SGA and hypoalbuminemia (≤3.1 g/dL) were associated with chemotherapy-induced side effects in lung cancer patients treated with paclitaxel and cisplatin [24]. However, covariates were not accounted in their analyses, and there was no significant association for side effects over grade 3. Also, it was not clear whether hypoalbuminemia was associated with albumin-binding characteristic of paclitaxel. Severe malnutrition assessed by NRI was shown to increase the adverse events and reduce the survival rate in metastasized colon cancer patients [25]. Especially, hypoalbuminemia and inflammatory status are shown to increase hematological adverse events. Another study suggested that higher PG-SGA score increased patients’ hospitalization [26]. These studies used one or two indices to predict nutritional status. Also, in most of the studies, the number of patients was less than 100 including those with high severity. The present study used stomach cancer patients with grade 2 and 3A tumors using the same treatment protocol to minimize variables possibly affecting the occurrence of adverse events. We also compared a few different assessment tools to evaluate nutritional status. Our study results clearly show that hypoalbuminemia is a sensitive index to predict chemotherapy-associated side effects including neutropenia, the most frequently occurring hematological side effect. However, no significant association was observed for albumin with non-hematological adverse event. A recent review have indicated that nutritional status determined by PG-SGA was associated with nausea and vomiting in a consistent manner although the authors mentioned that non-hematological adverse events such as nausea and vomiting possess subjective nature [27]. To secure the reliability and reduce the variability of medical records on non-hematological adverse events, only those recorded by internal medicine specialists sharing identical diagnostic standards were used.

In this study, PG-SGA did not show a significant association with either hematological or non-hematological adverse events. A most presumable reason for this null association is a lack of sensitivity represented by PG-SGA. Patients in the present study are those who are stable and in the course of transition to oral feeding after gastrectomy. Therefore, nutritional status could be overestimated through PG-SGA score. We also evaluated % weight change (weight loss) between the initial hospitalization and shortly before starting chemotherapy as a nutritional status index. Many of the patients have already lost their weight at the initial hospitalization, and this may explain the lack of sensitivity by % weight loss in chemotherapy-induced adverse events. Recently, Consensus Statement from the European Society of Clinical Nutrition and Metabolism (ESPEN) suggested to use either BMI less than 18.5 kg/m^2^ or the combination of weight loss together with either a low BMI or a low fat fess mass index as diagnostic criteria for malnutrition [28]. In the meantime, the American Society for Parenteral and Enteral Nutrition (ASPEN) suggested that two or more out of six characteristics including insufficient energy intake, weight loss, loss of muscle mass, loss of subcutanueou fat, localized of generalized fluid accumulation, diminished functional status as measured by hand-grip strength can be used to diagnose adult malnutrition [29]. Nutritional assessment based on these suggestions may better predict possible adverse events in these patients. Neutropenia was the second most frequently occurring adverse event. Female, total gastrectomy, and lower albumin concentration were significantly associated with neutropenia. A recent multicenter randomized controlled trial indicated that capecitabine plus oxaliplatin adjuvant therapy for D2 gastrectomy exhibited neutropenia in 60% of the enrolled subjects, and 3- year disease free survival was significantly higher in males but not in females indicating sex-specific treatment protocol may be required based on chemotherapy-induced adverse events [30].

Limitations of this study are as below. First, this is a retrospective study which may have pit-falls in the collected data. To minimize the limitation, we have included entire medical records written by internal medicine specialists during S-1 therapy. Secondly, the initial body weight used to determine % weight change was the measurement after gastrectomy, and therefore, removed tissue weight was not calculated. Lastly, only 16% of the patients were reported to have side-effects, and this may cause a lack of sensitivity to PG-SGA score and NRI.

## Conclusions

In conclusion, blood albumin concentration can be used as a marker of a successful completion of cancer chemotherapy before starting chemotherapeutic treatment. Findings of this study also suggest there is frequently used nutritional screening tools were unable to predict major chemotherapy-induced adverse events which suggest PG-SGA and % weight loss may not be sensitive indices to predict treatment endurance in these patients. Further studies on standardized nutrition screening with a larger number of cancer patients with specific type of cancer may provide accurate diagnostic value of nutritional screening tools applicable to different clinical settings.

## Additional files

Additional file 1: Table S1. Multivariable logistic regression analysis of risk factors for chemotherapy regimen. (DOCX 19 kb)
Additional file 2: Table S2. The relationships of PG-SGA and NRI with diarrhea and abdominal pain. (DOCX 12 kb)

## Abbreviations

BMI: Body Mass Index
NRI: Nutrition Risk Index
PG-SGA: Patient-Generated Subjective Global Assessment
