# Supplementary material for: Association of nutritional status-related indices and chemotherapy-induced adverse events in gastric cancer patients
Source: BMC Cancer. 2016 Nov 18;16:900. doi: 10.1186/s12885-016-2934-5 (PMC5116147; doi:10.1186/s12885-016-2934-5)
Supplement: Additional file 1: Table S1. — Multivariable logistic regression analysis of risk factors for chemotherapy regimen. (DOCX 19 kb) [file 12885_2016_2934_MOESM1_ESM.docx]

**Additional file: 1 Table S1. Multivariable logistic regression analysis of risk factors for chemotherapy regimen**

| Variables | | Chemotherapy completion | | | | Delayed chemotherapy | | | |
| --- | --- | --- | --- | --- | --- | --- | --- | --- | --- |
|  |  | Univariate | | Multivariate ^a)^ | | Univariate | | Multivariate ^b)^ | |
|  |  | OR (95% CI) | *p* | OR (95% CI) | *p* | OR (95% CI) | *p* | OR (95% CI) | *p* |
| Age (y) |  | 1.04(1.02-1.07) | 0.001 | 1.04(1.01-1.07) | 0.003 | 0.99(0.96-1.02) | 0.426 |  |  |
| Sex | Female | 0.51(2.78-0.94) | 0.031 | 0.57(0.31-1.07) | 0.082 | 1.75(0.92-3.33) | 0.086 |  |  |
|  | Male | 1.00 |  | 1.00 |  | 1.00 |  |  |  |
| Operation type | Total | 0.69(0.39-1.25) | 0.221 |  |  | 2.34(1.23-4.45) | 0.010 | 2.20(1.15-4.23) | 0.018 |
|  | Distal | 1.00 |  |  |  | 1.00 |  | 1.00 |  |
| Creatinine clearance (mL/min) ^c)^ | ≥60 | 1.00 | 0.490 |  |  | 1.00 | 0.794 |  |  |
|  | < 60 | 1.29(0.62-2.72) |  |  |  | 0.89(0.36-2.17) |  |  |  |
| Albumin  (g/dL) | <3.5 | 1.76(0.83-3.71) | 0.141 |  |  | 2.49(1.13-5.48) | 0.024 | 2.25(1.01-5.04)^†^ | 0.048 |
|  | ≥3.5 | 1.00 |  |  |  | 1.00 |  | 1.00 |  |
| BMI (kg/m^2^) |  | 1.05(0.94-1.16) | 0.406 |  |  | 0.99(0.89-1.12) | 0.976 |  |  |
| PG-SGA category | A | 1.00 | 0.930 |  |  | 1.00 | 0.630 |  |  |
|  | B | 1.03(0.55-1.91) |  |  |  | 0.84(.040-1.73) |  |  |  |
| PG-SGA score |  | 1.04(0.94-1.16) | 0.443 |  |  | 1.02(0.90-1.15) | 0.764 |  |  |
| Weight loss (%) |  | 0.96(0.88-1.04) | 0.268 |  |  | 0.46(0.88-1.06) | 0.967 |  |  |
| NRI |  | 0.99(0.94-1.04) | 0.626 |  |  | 0.93(0.88-0.99) | 0.019 | 0.94(0.89-4.05)^††^ | 0.055 |

^a)^ Covariates used in multivariate analyses included age and sex; ^b)^ Covariates used in multivariate analyses included operation type, albumin, and NRI; ^c)^ Creatinine clearance was calculated using the Cockroft–Gault formula; ^†^ NRI was not included in covariates due to the multicollinearity; ^††^ Albumin was not included in covariates due to the multicollinearity; BMI, Body Mass Index; PG-SGA, Patient-Generated Subjective Global Assessment; NRI, Nutritional Risk Index.
