# Supplementary material for: Association of nutritional status-related indices and chemotherapy-induced adverse events in gastric cancer patients
Source: BMC Cancer. 2016 Nov 18;16:900. doi: 10.1186/s12885-016-2934-5 (PMC5116147; doi:10.1186/s12885-016-2934-5)
Supplement: Additional file 2: Table S2. — The relationships of PG-SGA and NRI with diarrhea and abdominal pain. (DOCX 12 kb) [file 12885_2016_2934_MOESM2_ESM.docx]

**Additional file 2: Table S2. The relationships of PG-SGA and NRI with diarrhea and abdominal pain**

|  | | Univariate | | | |
| --- | --- | --- | --- | --- | --- |
| Variables | | Diarrhea | | Abdominal pain | |
|  |  | OR (95% CI) | *p* | OR (95% CI) | *p* |
| PG-SGA | SGA score <9 | 1.00 | 0.496 | 1.00 | 0.873 |
|  | SGA score ≥9 | 1.48(0.48-4.59) |  | 1.12(0.28-4.47) |  |
| NRI | Adequate | 1.00 | 0.131 | 1.00 | 0.357 |
|  | Malnutrition | 3.23(0.71-14.81) |  | 2.09(0.43-10.11) |  |

PG-SGA, Patient-Generated Subjective Global Assessment; NRI, Nutritional Risk Index.
